# Bioinformatics Analysis Identifies p53 as a Candidate Prognostic Biomarker for Neuropathic Pain

**DOI:** 10.3389/fgene.2018.00320

**Published:** 2018-08-31

**Authors:** Yibo Gao, Na Sun, Lieju Wang, Ying Wu, Longfei Ma, Juncong Hong, Jinxuan Ren, Bin Zhu, Lina Yu, Min Yan

**Affiliations:** ^1^Department of Anesthesiology, Second Affiliated Hospital of Zhejiang University School of Medicine, Hangzhou, China; ^2^Graduate School, Xuzhou Medical University, Xuzhou, China

**Keywords:** neuropathic pain, bioinformatics analysis, dorsal root ganglia, p53, caspase-3, chronic constriction injury, neuron apoptosis

## Abstract

Neuropathic pain (NP) is a type of chronic pain that is different from the common type of pain. The mechanisms of NP are still poorly understood. Exploring the key genes and neurobiological changes in NP could provide important diagnostic and treatment tools for clinicians. GSE24982 is an mRNA-seq dataset that we downloaded from the Gene Expression Omnibus database to identify key genes in NP. Differentially expressed genes (DEGs) were identified using the BRB-ArrayTools software and R. Functional and pathway enrichment analyses of the DEGs were performed using Metascape. A protein–protein interaction network was created and visualized using Cytoscape. A total of 123 upregulated DEGs were obtained. Among these genes, p53 was the node with the highest degree; hence, we validated it experimentally using a chronic constriction injury mouse model. Our results showed that overexpression of the p53 gene, and the subsequent increase in caspase-3 expression, in dorsal root ganglion neurons led to increased apoptotic changes in these neurons. p53 may therefore be partly responsible for the development of chronic constriction injury-induced NP.

## Introduction

Neuropathic pain (NP) is a kind of chronic pain caused by injury to the nervous system, and is characterized by dysesthesia, hyperalgesia, and allodynia (Finnerup et al., 2016). Its pathogenesis is complex, and there is still a lack of effective therapeutic drugs. Recent research has indicated that the gene expression of primary sensory nerves can be changed by peripheral nerve injury (von Hehn et al., 2012). Therefore, the study of gene expression in the dorsal horn of the spinal cord after peripheral nerve injury is very important to explore the mechanisms underlying NP and to provide an effective therapeutic strategy for NP.

Bioinformatics provides tools for the analysis of large-scale information to produce comprehensive overviews of genetic networks and potential biological pathways based on phenotypic patterns. This study aimed to explore the molecular mechanisms underlying NP after nerve injury and to identify key genes and potential pathways associated with the pathogenesis of NP. In this study, the GSE24982 mRNA-seq dataset, which contains the mRNA expression profile of a spinal nerve ligation (SNL) rat model of NP, was analyzed after downloading it from the Gene Expression Omnibus (GEO) database. Differentially expressed genes (DEGs) were identified, a functional enrichment analysis was carried out, and then a protein–protein interaction (PPI) network of upregulated DEGs was constructed.

Nerve system injury triggers a series of biochemical and cellular processes (Bareiss et al., 2015), including edema, axon fragmentation, electrolyte imbalance, demyelination, excitotoxicity, and endoplasmic reticulum stress, causing secondary injury and leading to neuronal apoptosis (Byrnes et al., 2007; Casha et al., 2012; Wu et al., 2015). Neuronal apoptosis may lead to a series of neurological impairments after spinal cord injury (SCI) (Choo et al., 2008; Niu and Yip, 2011). In this study, we predicted and analyzed the mRNAs in dorsal root ganglion (DRG) neurons that were involved in NP by using bioinformatics tools, and we found that p53 was the key gene. A chronic constriction injury (CCI)-induced NP mouse model was then established and the protein levels of p53 in the DRG neurons were detected. We verified that p53 was involved in nerve injury in DRG neurons, and it induced increased caspase-3 levels in these neurons after nerve injury. Moreover, the number of DRG neurons decreased significantly in the CCI group compared to the control group. In addition, a p53 inhibitor decreased p53 expression in the DRG neurons and reduced apoptosis, and it reduced the development of CCI-induced hyperalgesia in the mice. In conclusion, the p53–caspase-3 pathway plays a pivotal role in nerve injury in DRG neurons, and this pathway is related to apoptosis.

## Materials and Methods

### mRNA-seq Data

The GSE24982 dataset was downloaded from the GEO database. This dataset was based on the Affymetrix platform GPL1355 (Affymetrix Rat Genome 230 2.0 Array), and it was submitted by von Schack et al. (2011). The data in this dataset are based on eight groups of tissue samples comprising contralateral and ipsilateral L4 and L5 DRG tissues collected 4 weeks after the rats underwent L5 SNL or a sham operation (with no L5 SNL). The eight groups are as follows: (i) contralateral L4 DRG tissue from sham group mice (*n* = 5), (ii) ipsilateral L4 DRG tissue from sham group mice (*n* = 5), (iii) contralateral L4 DRG tissue from SNL group mice (*n* = 5), (iv) ipsilateral L4 DRG tissue from SNL group mice (*n* = 5), (v) contralateral L5 DRG tissue from sham group mice (*n* = 5), (vi) ipsilateral L5 DRG tissue from sham group mice (*n* = 5), (vii) contralateral L5 DRG tissue from SNL group mice (*n* = 5), and (viii) ipsilateral L5 DRG from SNL group mice (*n* = 5). Thus, there were 40 samples, comprising 20 SNL samples and 20 sham cohort samples.

### Identification of DEGs

The Affymetrix CEL raw data files were analyzed using the BRB-ArrayTools software (version 4.5.1 - Stable) and R (version 3.2.5). Because of the large number of probes (risking a high false positive rate), the eBayes algorithm provided in the Limma package (Phipson et al., 2016) was used. As an appropriate and conservative approach to identifying DEGs, the thresholds were set at | logFC|≥ 1.5 (where FC is the fold change) and an adjusted *P* < 0.05. Once the intensity values of the probes were <20%, they were filtered out using the “Filter Probe Sets by Expression” option. Hierarchical clustering analysis was used to categorize the data.

### DEG Gene Ontology (GO) Analysis and Pathway Enrichment

Gene Ontology (GO) analysis is a major bioinformatics tool for annotating genes and gene products (often for interpreting high-throughput genome or transcriptome data), and it aims to unify the representation of gene and gene product attributes across all species (Ashburner et al., 2000; Gene Ontology, 2006). The Kyoto Encyclopedia of Genes and Genomes (KEGG^1^) is a collection of databases containing advanced functional information for the systematic analysis of gene functions, biological pathways, diseases, drugs, and chemical substances (Kanehisa and Goto, 2000). Pathway and process enrichment analyses were carried out with the above sources.

### PPI Network

Protein–protein interaction enrichment analysis was carried out using the following databases: BioGRID (Stark et al., 2006), InWeb_IM (Li et al., 2017), and OmniPath (Turei et al., 2016). Metascape^2^ is a free online resource with an automated meta-analysis tool for understanding the biological significance of a large number of genes (Tripathi et al., 2015). Metascape and Cytoscape software (version 3.5.1) (Shannon et al., 2003) were used to build the PPI network, and a confidence score >0.7 was set as the cut-off criterion. Molecular Complex Detection (MCODE) (Bader and Hogue, 2003) was used to screen the modules of the PPI network using the following parameters: degree cut-off = 2, node score cut-off = 0.2, *k*-core = 2, and maximum depth = 100 (Ashburner et al., 2000; Wang et al., 2018).

### Animal Experiments

This study was carried out in accordance with the recommendations of the International Association for the Study of Pain. The protocol was approved by the Zhejiang Animal Care and Use Committee and the Ethics Committee of the Second Affiliated Hospital of Zhejiang University. All animal experiments were performed on adult male C57BL/6 mice (weighing 18–20 g), which were purchased from the Experimental Animal Center of Zhejiang University. The animals were kept in cages under controlled conditions, with the room temperature fixed at 23 ± 1°C, a 12 h/12 h light/dark cycle, and food and water available *ad libitum*. All animals were left to acclimate for 1 week before any experimentation.

### Mouse Model of NP

Under the protocol described by Bennett and Xie, NP was induced by sciatic nerve CCI (Bennett and Xie, 1988). The mice were anesthetized using sodium pentobarbital (40 mg/kg) via intraperitoneal injection. The left leg of each mouse was shaved and the skin was sterilized. The skin was incised at the mid-thigh level and the muscles were separated to expose the left sciatic nerve. The sciatic nerve was tied loosely with four ligatures of silk thread (4-0) spaced at 1 mm. The sham surgery (without ligation) was performed in the same manner, i.e., the left sciatic nerve was exposed. Finally, the muscles and skin were sutured and sterilized. The mice were then allowed to recover in their cages.

### Intrathecal Injections of Pifithrin-α

Intrathecal injections of the p53 inhibitor pifithrin-α (HY-15484; MedChemExpress, United States) were administered as described by Mestre et al. (1994). In short, the mice were anesthetized using sodium pentobarbital and a 25-μL Hamilton syringe of pifithrin-α was then inserted into the subarachnoid cavity via the vertebral column between the L4 and L5 vertebrae. The stereotypical tail flick reflex (i.e., a sudden lateral movement of the tail) indicating that the needle had entered the subarachnoid cavity was used to confirm successful placement. In the CCI + pifithrin-α group, pifithrin-α was administered postoperatively at a dose of 10 μg (in a volume of 10 μl) once a day for 3 days. The same procedure was performed for the CCI + DMSO group, with an injection of 10 μl 4% dimethyl sulfoxide (DMSO) instead of pifithrin-α.

### Thermal Hyperalgesia Assessment

To quantify thermal hyperalgesia in the mice, paw withdrawal latency (PWL) was assessed using the procedure described by Hargreaves et al. (1988) with a plantar analgesia meter (model 390; IITC Life Science). The mice were placed separately into acrylic cages and left to adapt to the environment for half an hour before the test. The plantar surface of each mouse’s left hind paw was exposed to a radiant heat source through a glass plate. Once the mice exhibited a paw retract or paw licking reaction, the radiant heat source was immediately turned off, and the total irradiating time was recorded as the PWL value. If the mouse did not have a paw retract or paw licking reaction within 20 s, the radiant heat source was turned off to protect the mouse tissue from burns, and “20 s” was recorded as the PWL value. Three measurements were performed for each mouse, and the mean PWL was taken as the final PWL. PWL testing was conducted for all mice 3 days before surgery and 1, 3, 5, and 7 days postoperatively.

### Immunofluorescence Assays

For immunofluorescence, DRG sections were incubated at 4°C overnight with a mixture of mouse anti-NeuN antibody (which targets NeuN, a neuron marker; MAB377; 1:200; Merck Millipore) and either mouse anti-p53 antibody (1C12; 1:200; Cell Signaling Technology) or rabbit anti-cleaved-caspase-3 antibody (ASP175; 1:200; Cell Signaling Technology). After washing in phosphate-buffered saline (PBS), the sections were incubated with a mixture of fluorescein-conjugated donkey anti-rabbit secondary antibody (A0453; 1:500; Beyotime) and fluorescein-conjugated goat anti-mouse secondary antibody (A0428; 1:500; Beyotime) for 1 h at room temperature away from light. The sections were washed again and dried. Finally, coverslips were placed on the slides with Gel Mount aqueous mounting medium. Images were captured using fluorescence microscopy (Olympus).

### Western Blotting

Each mouse was sacrificed after anesthetizing it with sodium pentobarbital. The DRG neurons were rapidly dissected out and homogenized for 30 min on ice in lysis buffer containing protease inhibitors. The tissue lysates were centrifuged at 13,000 rpm for 10 min at 4°C and the supernatants were collected. The protein concentration was estimated according to the Bradford method (Bradford, 1976). After being heated at 100°C for 5 min and mixed with 1 × loading buffer, equal amounts of protein were separately loaded onto and separated on 10% sodium dodecyl sulfate polyacrylamide gel electrophoresis (SDS–PAGE) gels. The separated proteins were transferred onto polyvinylidene difluoride (PVDF) membranes. The membranes were blocked in 10% non-fat milk for 1 h at room temperature. The membranes were then incubated overnight at 4°C with rabbit anti-cleaved-caspase-3 primary antibody (ASP175; 1:1000; Cell Signaling Technology), mouse anti-p53 primary antibody (1C12; 1:1000; Cell Signaling Technology), or mouse anti-glyceraldehyde 3-phosphate dehydrogenase (GAPDH) antibody (60004-1-ig; 1:1000; Proteintech Group, Rosemont, IL, United States). After washing in Tris-buffered saline with Tween-20 (TBST), the membranes were incubated with secondary antibody: horseradish peroxidase (HRP)-conjugated goat anti-rabbit antibody (A0208; 1:5000; Beyotime) or HRP-conjugated goat anti-mouse antibody (A0216; 1:5000; Beyotime) at room temperature for 1 h. After incubation, the membranes were washed again with TBST. Quantity One 4.6.2 (Bio-Rad, United States) was used to analyze the western blot density values.

### Statistical Analyses

Statistical analyses were performed using GraphPad Prism 6 (GraphPad Software, Inc., San Diego, CA, United States). Student’s *t*-test was used to compare the data between pairs of groups. Two-way repeated-measures analysis of variance (ANOVA) followed by *post hoc* tests using the Bonferroni correction was employed to compare the data among more than two groups. All values are expressed as mean ± standard deviation (SD). *P* < 0.05 was considered statistically significant.

## Results

### Data Normalization

**FIGURE 1:**
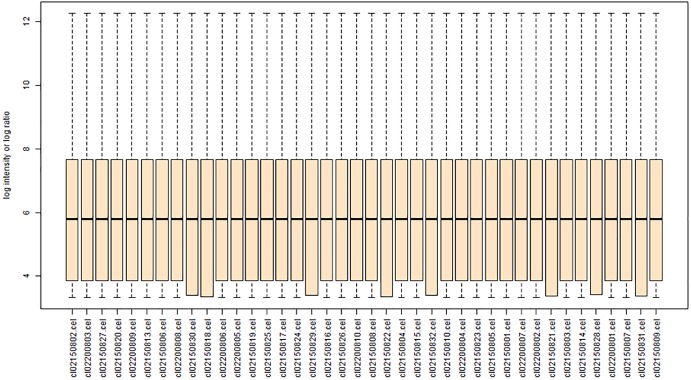
Boxplot showing the gene expression of each rat DRG tissue sample [contralateral or ipsilateral L4 or L5 tissue after L5 spinal nerve ligation (SNL) or sham surgery] after data normalization. The ordinate represents the gene expression values of each sample, and the abscissa represents SNL samples and sham cohort samples. The horizontal lines in each column represent the mean gene expression value.

Each array was normalized (centered) by quantile normalization using the BRB-ArrayTools software. Genes were excluded if <20% of the expression data had ≥1.5-fold change (in either direction) compared to the gene’s median value or the missing or filtered out data was >50%. **Figure 1** shows a boxplot of gene expression in each of the eight groups in the GSE24982 dataset.

### Identification of DEGs

**FIGURE 2:**
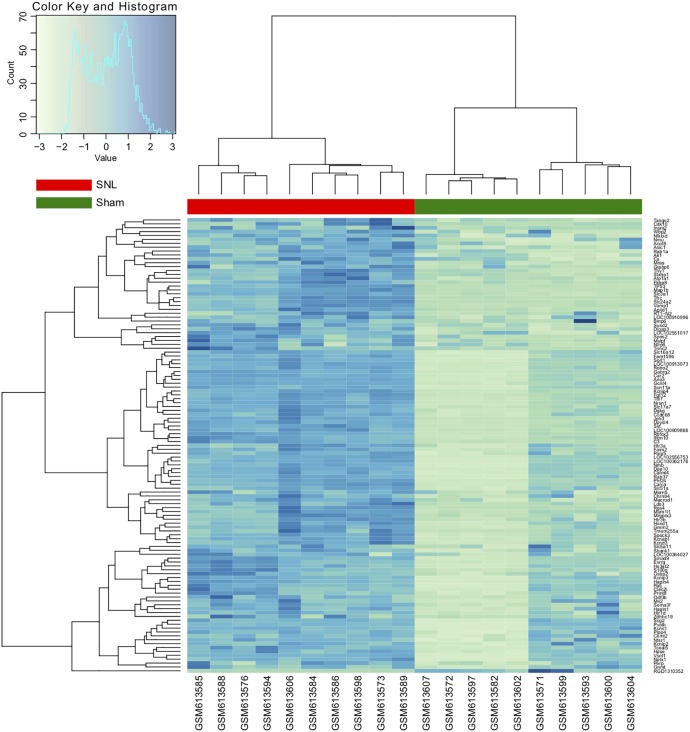
Hierarchical clustering analysis of differentially expressed genes (DEGs). Each row represents a single gene; each column represents a sample. Clustering distance is based on the Euclidean distance.

In NP models, mechanical allodynia and thermal withdrawal latency are usually significantly decreased in the ipsilateral hind paw, and the relative protein expression levels change, but not on the contralateral side. Therefore, in this study, we selected the ipsilateral L4 and L5 DRG tissues to identify the DEGs. A total of 22,303 genes were identified from the GSE24982 dataset, and among them, 123 genes were upregulated in DRG neurons in the SNL group compared to the sham cohort (**Supplementary Table S1**). Hierarchical clustering analysis was used to categorize the genes with similar expression patterns into two groups (**Figure 2** and **Supplementary Table S1**).

### Pathway and Process Enrichment Analyses

**FIGURE 3:**
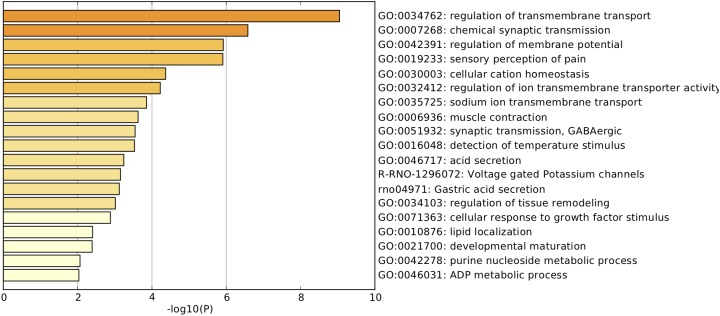
Pathway and process enrichment analysis. Heatmap of enriched terms related to the inputted list of genes, colored according to their *p*-values.

Pathway and process enrichment analyses were carried out with the following ontology sources: KEGG pathways and GO biological processes. All genes in the genome were used as the enrichment background. Terms with *P* < 0.01, minimum count = 3, and enrichment factor (ratio between observed count and count expected by chance) >1.5 were collected and grouped into clusters based on membership similarities. More specifically, *p*-values were calculated based on accumulative hypergeometric distribution, *q*-values were calculated using the Benjamini–Hochberg procedure to account for multiple testing (Hochberg and Benjamini, 1990), Kappa scores were used as the similarity metric when performing hierarchical clustering of the enriched terms, and subtrees with similarity >0.3 were considered a cluster. Regarding the hierarchical clustering of the enriched pathway and process terms, the most statistically significant term within a cluster was selected for cluster annotation (**Figure 3** and **Supplementary Table S2**). We found that the upregulated DEGs were mainly associated with terms related to the regulation of transmembrane transport, chemical synaptic transmission, regulation of membrane potential, sensory perception of pain, cellular cation homeostasis, regulation of ion transmembrane transporter activity, sodium ion transmembrane transport, voltage-gated potassium channels, cellular response to growth factor stimulus, and developmental maturation.

### PPI Network Analysis

**FIGURE 4:**
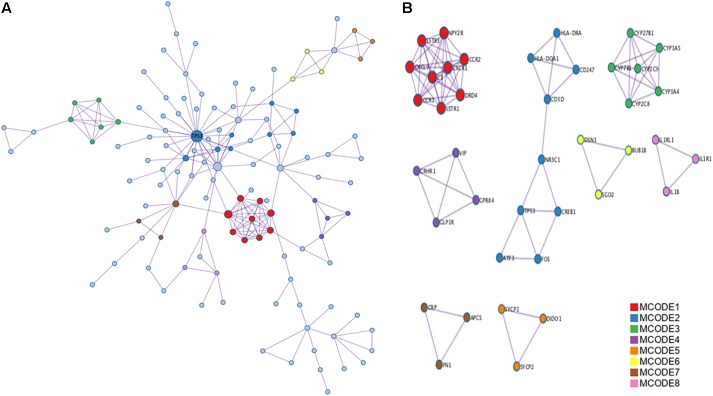
Protein–protein interaction (PPI) network and Molecular Complex Detection (MCODE) components identified related to the upregulated differentially expressed genes (DEGs). **(A)** PPI network of proteins encoded by DEGs. **(B)** Modules selected from PPI network using MCODE. Degrees >6 was set as the cut-off criterion. Nodes represent upregulated DEGs; lines represent interaction relationships between nodes.

A PPI network analysis of the proteins encoded by the identified DEGs was carried out using the following databases: BioGRID, InWeb_IM, and OmniPath. Metascape and Cytoscape software were used to build the PPI network (**Figure 4A**). The resultant network contained the subset of proteins that formed physical interactions with at least one other protein encoded by a DEG. The study protocol indicated that if the PPI network contained 3–500 proteins, MCODE was to be used to identify densely connected network components. Significant modules (and the associated individual genes) in the PPI network were identified using MCODE (**Figure 4B**, MCODE1 and MCODE2 modules), and p53 was identified as the hub gene.

### Behavioral Changes After CCI

**FIGURE 5:**
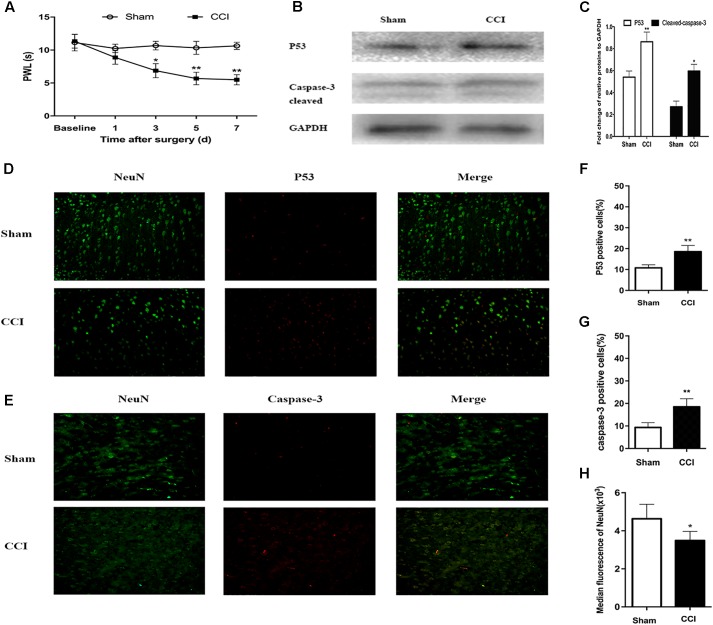
Chronic constriction injury (CCI)-induced hyperalgesia and expression of p53 and caspase-3 in dorsal root ganglion (DRG) neurons. **(A)** Compared to the sham group mice, the CCI mice had decreased paw withdrawal latency (PWL) from day 3 after surgery. **(B)** Western blot analysis of the expression of p53 and caspase-3 on day 7 after CCI or sham surgery. The fold change of p53 and caspase-3 was normalized to the glyceraldehyde 3-phosphate dehydrogenase (GAPDH) level. **(C)** Quantification of p53 and caspase-3 expression. Double immunofluorescence staining for p53 and caspase-3 in DRG neurons. **(D)** Representative immunofluorescence staining of p53 (red) and its colocalization with neurons (NeuN, green) in DRG tissues. **(E)** Representative immunofluorescence staining of caspase-3 (red) and its colocalization with neurons (NeuN, green) in DRG tissues. **(F,G)** Quantitative analysis of p53-positive and caspase-3-positive cells (%) in DRG tissues at 7 days after CCI. **(H)** Median fluorescence of NeuN. Magnification: 100× for all columns. Data are all expressed as mean ± SD. ^∗^*P* < 0.05, ^∗∗^*P* < 0.01, compared with the sham group; *n* = 6 in each group.

Research has shown that CCI in mice induces spontaneous pain behaviors, persistent allodynia, and hyperalgesia in the affected paw (Dowdall et al., 2005). As shown in **Figure 5A**, on day 3 postoperatively, the CCI mice had a significantly lower threshold for thermal hyperalgesia (as indicated by a shorter PWL) compared with the sham mice (6.87 ± 0.53 s vs. 10.67 ± 3.7 s; *P* < 0.05), and this difference was maintained on day 7 after surgery (5.50 ± 0.4 vs. 10.64 ± 1.14; *P* < 0.01).

### Detection of p53 and Caspase-3 Expression in CCI Mice by Western Blot and Immunofluorescence Assays

The PPI analysis indicated that p53 was the node with the highest degree. Therefore, firstly, we sought to confirm whether CCI can induce p53 expression in DRG neurons. Compared with the sham group mice, the p53 level in the CCI mice was significantly higher according to the western blot analysis (*P* < 0.01; **Figure 5B**). Furthermore, when assessing the release of active caspase-3, cleaved caspase-3 expression was found to be significantly higher in the CCI group than that in the sham group (*P* < 0.05; **Figure 5C**).

According to the immunofluorescence assays, p53 and caspase-3 were highly expressed in the ipsilateral DRG tissues after CCI, but they were rarely found in the corresponding sham group (*P* < 0.01; **Figures 5D–G**). In addition, the number of DRG neurons decreased by 1/4 in the CCI mice compared with the sham group mice on day 7 post-surgery (*P* < 0.05; **Figures 5D,E,H**). These findings reveal that ligation of the sciatic nerve leads to an increase in p53 and caspase-3 expression, along with a decrease in the number of DRG neurons.

### p53 Inhibitor Reduced p53 Protein Expression and Attenuated Thermal Hyperalgesia in CCI Mice

**FIGURE 6:**
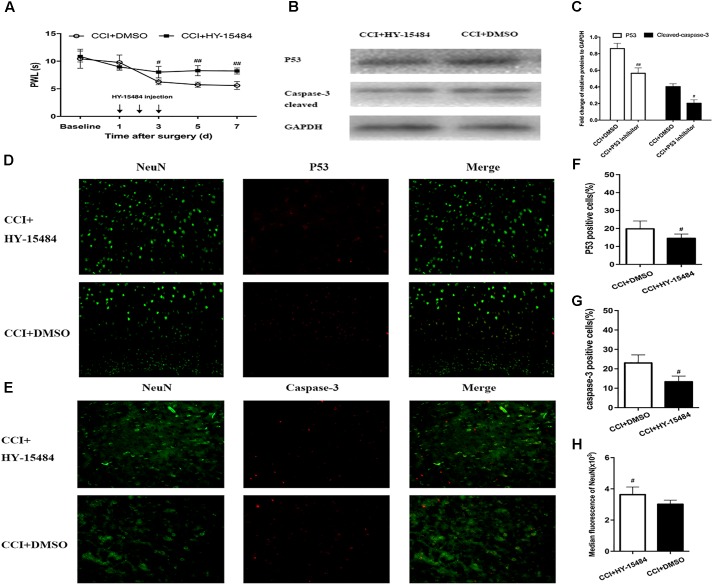
Inhibition of p53 by pifithrin-α (HY-15484) restrained the pain behaviors and expression of p53 and caspase-3 in dorsal root ganglion (DRG) neurons. **(A)** Thermal hyperalgesia after intrathecal injection of pifithrin-α (10 μg). Pifithrin-α attenuated chronic constriction injury (CCI)-induced thermal hyperalgesia, compared with that in the CCI + DMSO group. **(B)** Western blot analysis of the expression of p53 and caspase-3 after intrathecal administration of pifithrin-α on day 7 after CCI. The fold change of p53 and caspase-3 levels was normalized to the glyceraldehyde 3-phosphate dehydrogenase (GAPDH) level. **(C)** Quantification of p53 and caspase-3 expression. Double immunofluorescence staining for p53 and caspase-3 in DRG neurons after intrathecal administration of pifithrin-α on day 7 after CCI. **(D)** Representative immunofluorescence staining of p53 (red) and its colocalization with neurons (NeuN, green) in DRG tissues. **(E)** Representative immunofluorescence staining of caspase-3 (red) and its colocalization with neurons (NeuN, green) in DRG tissues. **(F,G)** Quantitative analysis of p53-positive and caspase-3-positive cells (%) in DRG tissues at 7 days after intrathecal administration of pifithrin-α in the CCI model. **(H)** Median fluorescence of NeuN. Magnification: 100× for all columns. Data are all expressed as mean ± SD. ^#^*P* < 0.05, ^##^*P* < 0.01, compared with the CCI + DMSO group; *n* = 6 in each group.

After treatment with pifithrin-α (CCI + pifithrin-α mice), the threshold for thermal hyperalgesia (as indicated by a shorter PWL) was significantly increased on day 3 after surgery compared to the threshold in the CCI + DMSO mice (8.02 ± 1.75 s vs. 6.21 ± 0.55 s; *P* < 0.05). This difference was maintained on day 7 after surgery (8.22 ± 2.62 s vs. 5.59 ± 0.54 s; *P* < 0.01) (**Figure 6A**). Significant differences were also observed in the number of p53-positive and caspase-3-positive neurons between the CCI + pifithrin-α and CCI + DMSO groups on day 7 after surgery (*P* < 0.05; **Figures 6D–G**). Western blot analysis revealed that the expression levels of p53 and cleaved caspase-3 on day 7 after CCI were significantly higher in CCI + DMSO mice than in CCI + pifithrin-α mice (*P* < 0.01; **Figures 6B,C**). The number of DRG neurons increased significantly in the CCI + pifithrin-α group compared to the CCI + DMSO group (**Figures 6D,E,H**). These findings reveal that the p53 inhibitor attenuated thermal hyperalgesia and decreased p53 and caspase-3 expression in CCI mice, and it was associated with a higher number of DRG neurons.

## Discussion

Neuropathic pain is caused by injury of the nervous system, leading to abnormal pain. Conventional analgesics often have poor treatment effects, and hence it is particularly important for researchers to discover more effective medications and other treatments. As the use of molecular biology techniques has advanced, the role of pain-related genes in the development of NP has become a key research topic. Research has shown that NP is often accompanied by changes in the expression of genes in the sensory pathway (von Hehn et al., 2012). The expression levels of thousands of genes in biological samples can be quantified simultaneously, and the whole-genome expression data obtained from these techniques allows the investigation of complex regulatory relationships between genes and may help us find better therapeutic targets for treating NP. In this study, GSE24982 mRNA-seq data were downloaded and analyzed, and 123 genes were found to be upregulated in the SNL group compared to the sham group.

NP is associated with structural and functional alterations in neurons, including long-term potentiation of synaptic transmission, new dendritic spine formation, increases in intrinsic cellular excitability, and ion channel rearrangements (Blom et al., 2014; Santello and Nevian, 2015; Kim et al., 2016). Ion channels, such as sodium, potassium, calcium, and acid-sensitive ion channels, play important roles in the occurrence and development of NP. In this study, according to the pathway and process enrichment analyses, the upregulated DEGs were mainly associated with the regulation of transmembrane transport, chemical synaptic transmission, regulation of membrane potential, sensory perception of pain, cellular cation homeostasis, regulation of ion transmembrane transporter activity, sodium ion transmembrane transport, voltage-gated potassium channels, cellular response to growth factor stimulus, and developmental maturation. Most of these pathways/processes are related to the biological processes underlying NP.

Von Schack et al. (2011) the group that originally submitted the GSE24982 dataset, performed an miRNA expression profiling study of DRG neurons in an SNL rat model. They used a combination of bioinformatics and experimental approaches (including mRNA microarray profiling comparisons between SNL and Sham group mice) to identify the biological functions affected by the observed changes in miRNA expression. Wang et al. (2018) used almost the same strategy in their SCI-induced NP bioinformatics study. They identified four targeted miRNAs (mir-204-5p, mir-519d-3p, mir-20b-5p, and mir-6838-5p), which are potential biomarkers for SCI patients. Inflammatory reactions and apoptosis are closely related. Apoptosis of dorsal horn neurons is potentially linked to the emergence of persistent pain. A loss of these neurons after nerve injury has been observed in previous research (Scholz et al., 2005). Inquimbert et al. (2018) also reported that *N*-methyl-D-aspartate receptor (NMDAR)-mediated neurodegeneration contributes to the development of chronic NP. The p53 signaling pathway is the most commonly mutated pathway in tumorigenesis (Bode and Dong, 2004). p53 can respond to DNA damage, various types of genotoxicity, and cytotoxic stress by inducing cell-cycle arrest and apoptosis (Tanaka et al., 2000). Nerve injury results in many molecular and cellular events, which in turn can produce inflammatory reactive gliomas and neuronal apoptosis (Choi et al., 2010; Zhang et al., 2012). In this study, we built a PPI network of proteins encoded by upregulated DEGs, and MCODE was used to identify densely connected network components. p53 was selected as the hub gene.

In the past few years, studies have found that apoptosis may play an important role in NP. For example, after sciatic nerve transection in an SCI model, Coggeshall et al. (2001) found that the number of neurons decreased in the ipsilateral dorsal horn. In a CCI rat model, de Novellis et al. (2004) found that the number of neurons on both sides of the spinal cord decreased by >50%. In this model, neuronal apoptotic pathways in the spinal cord were activated, but blocking the mGluR5 glutamate receptors in the spinal cord prevented the early overexpression of apoptosis-related genes and the morphological changes of the dorsal horn (de Novellis et al., 2004). Moore et al. (2002) confirmed that the inhibitory neurons involved in excitotoxicity-related death of spinal dorsal horn cells contribute to NP. Cobos et al. (2018) used a weighted gene coexpression network analysis (WGCNA) to assay changes in global gene expression in DRG neurons at 10 days after spared nerve injury, i.e., in a model of partial nerve injury. They constructed a PPI network consisting of 310 nodes and 442 edges, revealing key signaling molecules such as ATF3, JUN, BDNF, and p53. Maj et al. (2017) reported that cisplatin rapidly increases mitochondrial p53 accumulation in DRG neurons in the spinal cord and peripheral nerves without evidence of apoptosis. Cisplatin also reduced the mitochondrial membrane potential and led to abnormal mitochondrial morphology and impaired mitochondrial function in the DRG neurons (Maj et al., 2017). Caspase-3-mediated neuronal apoptosis has been shown to play an important role in NP maintenance, and caspase-3 inhibitors have been shown to prevent the development of SNL-induced hyperalgesia and allodynia (Joseph and Levine, 2004; Scholz et al., 2005). Moreover, Joseph and Levine (2004) showed that before neuronal apoptosis, the caspase signaling pathway was activated by inflammatory/immune mediators and contributed to the pain in small-fiber peripheral neuropathy. The results of the above studies show that the increased apoptotic changes in DRG neurons mediated by p53 and caspase-3 may play a role in the development of NP.

The CCI rat model is a mature model that was established by Bennett and Xie (1988), and results in mechanical allodynia and thermal hyperalgesia, and spontaneous pain symptoms that last >2 months (Bennett and Xie, 1988; Scholz et al., 2005). The SNL rat model was developed by Kim and Chung (1992), and it involves thermal hyperalgesia and mechanical allodynia accompanied by spontaneous pain lasting ≥4 months (Choi et al., 1994). Regarding the CCI and SNL models, the SNL model requires more difficult and invasive surgery and is associated with increased risk of lack of response and tissue and muscle injury. Therefore, we investigated the expression of p53 in a CCI mouse model. In the present study, thermal hyperalgesia developed 3 days after surgery and continued to day 7 after surgery (the last day on which the thermal hyperalgesia test was carried out). We investigated the expression of apoptosis-related proteins (p53 and active caspase-3) to determine whether or not apoptosis occurred after injury. In the ipsilateral DRG tissues of CCI mice, we ascertained, with immunofluorescence double-labeling, that the increased levels of p53 and caspase-3 were colocalized with NeuN expression. Western blotting showed a significant increase in the expression of p53, which occurred in parallel with an increase in the expression of active caspase-3. Our results also demonstrate that the number of ipsilateral L4–L5 DRG neurons decreased. The selective p53 antagonist, pifithrin-α, reduced the development of thermal hyperalgesia and led to decreased p53 and caspase-3 expression, consistent with lower apoptotic levels among DRG neurons. This provides further verification that apoptotic processes are involved in NP.

Clarifying the molecular etiology of nerve injury-induced neurodegeneration is essential. Our results showed that p53 and active caspase-3 were significantly upregulated after CCI, and significant numbers of DRG neurons died after CCI, demonstrating that overexpression of p53 may mediate DRG neuronal apoptosis. After treatment with pifithrin-α in CCI mice, p53 and active caspase-3 expression levels were downregulated, consistent with lower apoptotic levels among DRG neurons. We conclude that p53-mediated neuronal death may contribute to the development of chronic NP, and neuroprotection may offer a potential treatment strategy for NP.

## Author Contributions

YG constructed the mouse model, collected data, performed bioinformatics analysis, and wrote the first draft of the manuscript. NS was responsible for the immunofluorescence and western blotting assays. LW, YW, and LY performed the statistical analysis. JR and BZ were responsible for tissue collection. LM and JH performed the mouse behavior tests. All authors contributed to manuscript revision and read and approved the submitted version.

## Conflict of Interest Statement

The authors declare that the research was conducted in the absence of any commercial or financial relationships that could be construed as a potential conflict of interest.

## Footnotes

1 http://www.genome.jp/

2 http://metascape.org
